# Forces required to dynamize sliding screws in gamma nail and selfdynamizable internal fixator

**DOI:** 10.1186/s12891-024-07392-3

**Published:** 2024-04-08

**Authors:** Milan M Mitkovic, Nikola D Korunovic, Sasa S Milenkovic, Predrag M Stojiljkovic, Miodrag T Manic, Miroslav D Trajanovic

**Affiliations:** 1 Clinic for Orthopaedics and Traumatology “Academician Prof. Dr. Milorad Mitkovic”, University Clinical Center Nis, Nis, Serbia; 2 Faculty of Medicine, University of Nis, Nis, Serbia; 3 Faculty of Mechanical Engineering, University of Nis, Nis, Serbia

**Keywords:** Dynamization, Lag screw, Pertrochanteric fracture, Gait

## Abstract

**Background:**

Single limb support phase of the gait-cycle in patients who are treated for a pertrochanteric fracture is characterized by transversal loads acting on the lag screw, tending to block its dynamization. If the simultaneous axial force overcomes transversal loads of the sliding screw, the dynamization can still occur.

**Methods:**

Biomechanical investigation was performed for three types of dynamic implants: Gamma Nail, and two types of Selfdynamizable Internal Fixators (SIF) – SIF-7 (containing two 7 mm non-cannulated sliding screws), and SIF-10 (containing one 10 mm cannulated sliding screw). Contact surface between the stem and the sliding screws is larger in SIF implants than in Gamma Nail, as the stem of Gamma Nail is hollow. A special testing device was designed for this study to provide simultaneous application of a controlled sliding screws bending moment and a controlled transversal load on sliding screws (Q_t_) without using of weights. Using each of the implants, axial forces required to initiate sliding screws dynamization (Q_a_) were applied and measured using a tensile testing machine, for several values of sliding screws bending moment. Standard least-squares method was used to present the results through the linear regression model.

**Results:**

Positive correlation between Q_t_ and Q_a_ was confirmed (*p* < 0.05). While performing higher bending moments in all the tested implants, Q_a_ was higher than it could be provided by the body weight. It was the highest in Gamma Nail, and the lowest in SIF-10.

**Conclusions:**

A larger contact surface between a sliding screw and stem results in lower forces required to initiate dynamization of a sliding screw. Patients treated for a pertrochanteric fracture by a sliding screw internal fixation who have longer femoral neck or higher body weight could have different programme of early postoperative rehabilitation than lighter patients or patients with shorter femoral neck.

## Introduction

Pertrochanteric fractures, defined as trochanteric fractures with a fracture gap extending from the greater to the lesser trochanter, are widely treated by an internal fixation that includes a sliding component for femoral neck and head, angled to the implant stem between 120 and 140-degrees [1–3]. Sliding component is designed in the form of a sliding screw or a sliding blade [4–6]. Sliding feature of the component provides biomechanical loads (body weight and muscles tension) to result in translational pertochanteric fracture compression while maintaining the appropriate femoral neck-shaft angle, and implant relief [6, 7]. Any controlled movement of bone fragments aiming to a better fracture healing, including the translation mentioned above, is called dynamization [8, 9]. In some cases, which cannot be recognized in advance with certainty, absence of the dynamization may lead to mechanical complications, such as cut-out, implant breakage or fixation failure [10–12]. The compression between fracture fragments, as a result of the dynamization, is considered an important promoting factor in the process of a new bone remodelling to a morphological structure being most resistant to the local biomechanical forces [13, 14]. 

**Fig. 1:**
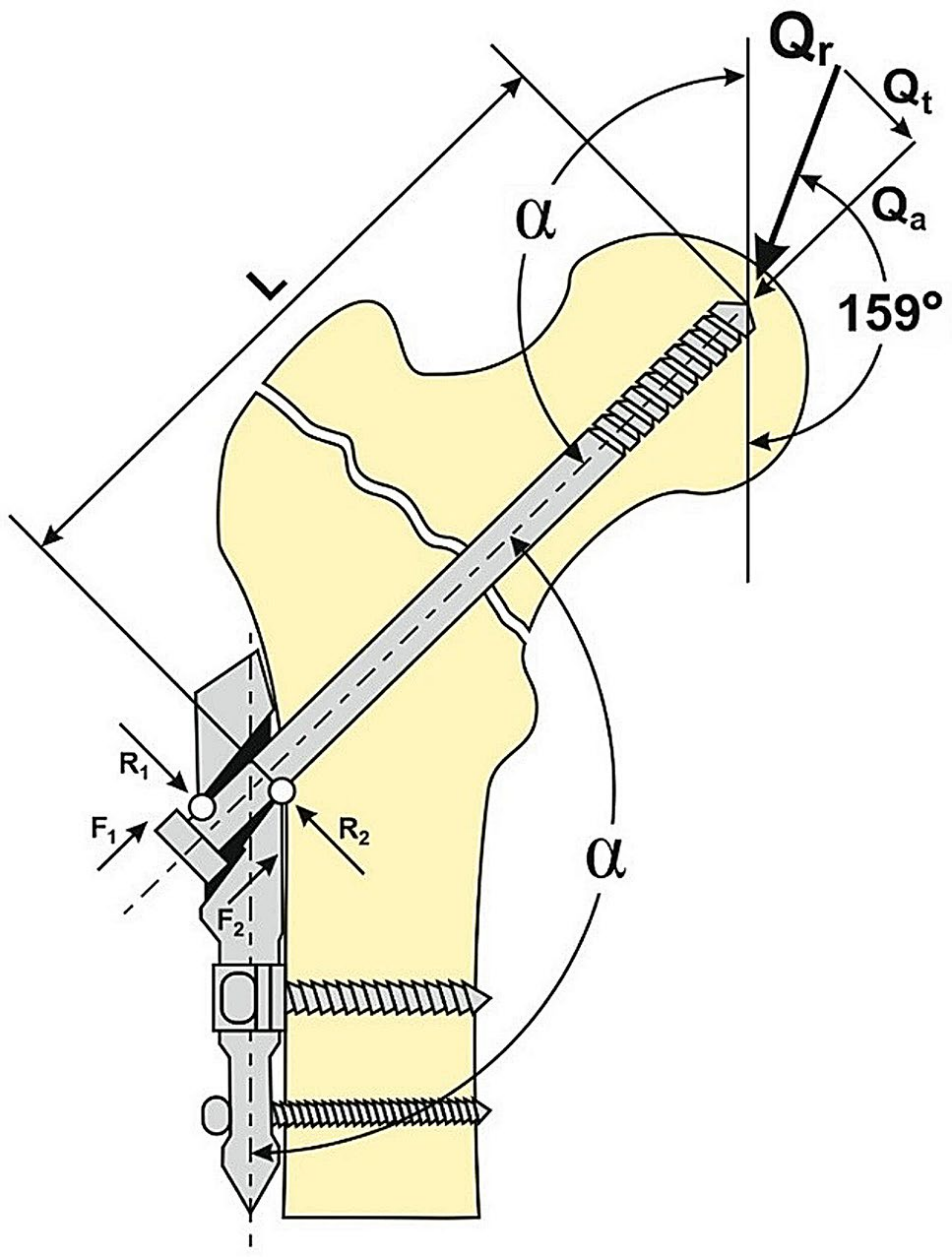
Diagram of single stance loads in pertrochanteric fracture fixed by a sliding screw implant. (Q_r_) Resulting force of the body weight; (Q_t_) transversal load of the sliding screw; (Q_a_) axial load of the sliding screw; (α) sliding screw-stem angle; (L) sliding screw extension length; (R_1_ and R_2_) reaction forces; and (F_1_ and F_2_) frictional forces

Single limb support phase accounts for about 40% of the gait-cycle [15, 16]. In this phase, hip joint endures the highest load due to the contraction of the abductor muscles aimed on maintaining the vertical body posture [7, 17]. Therefore, the compression of a fixed pertrochanteric fracture, that follows the dynamization of an implant sliding component, is expected to be highest during the ipsilateral single limb support. However, the load lever conditions prevailing at such moment can result as a breaking factor on the implant sliding component, having a potential to interfere with the dynamization [7, 18]. If the angle between the sliding component and the implant stem (α) is smaller, this self-locking effect is more likely to occur (Fig. 1).

Pertrochanteric fractures occur in the part of femur where the cancellous bone is dominant, and where it is bounded mainly by the thinned cortical bone of greater trochanter, thus fracture healing is accompanied mainly by inter-trabecular membranous bone formation [19, 20]. Inter-trabecular fracture healing can be a relatively rapid process, but it is spatially limited. This happens because cancellous bone formation after trauma is accompanied by simultaneous osteoid formation throughout the entire volume of traumatized tissue volume, but rarely extends more than 2 mm from the traumatized area [19]. This could be considered the reason why the dynamization feature is desirable in an internal fixation of pertrochanteric fractures surgical treatment. Excessive distance between cancellous fracture surfaces can be present either due to the fixed fracture position, or after initial osteolysis following the fracture fixation, being the risk for delayed or absent fracture healing. Maintaining contact between the fracture fragments can be ensured by the dynamization feature of the sliding screws. Accordingly, the objective of this study was to examine the forces required to initiate the dynamization of sliding screws in third generation Gamma Nail and in two types of Selfdynamizable Internal Fixator. Authors also wanted to present an original testing device that can be used in further similar examinations.

## Materials and methods

**Fig. 2:**
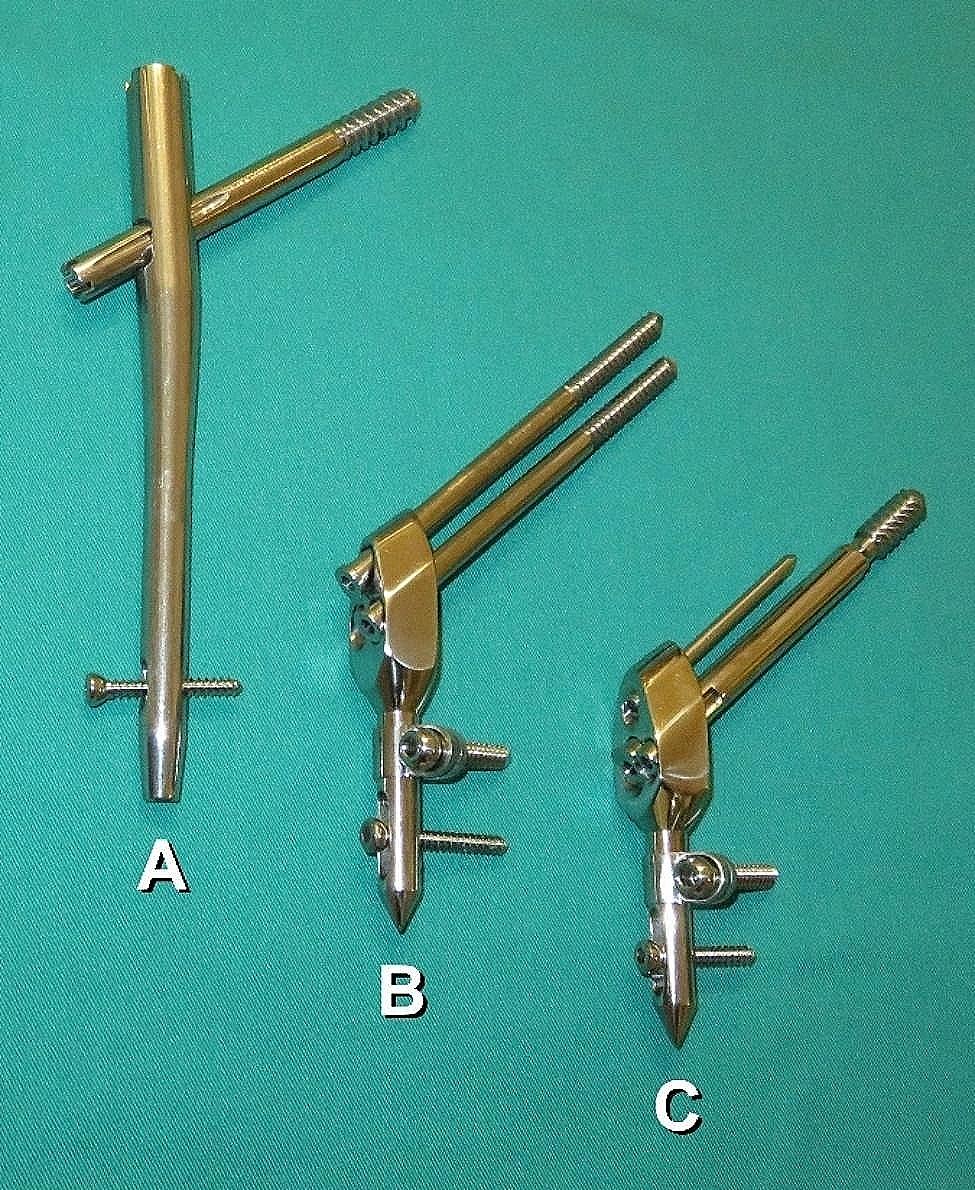
Implants tested in the study. (**A**) Gamma nail (Gamma3); (**B**) Selfdynamizable Internal Fixator with two sliding screws of 7 mm radius (SIF-7); and (**C**) Selfdynamizable Internal Fixator with one sliding screw of 10 mm radius and one locking anti-rotation pin (SIF-10)

Three types of implants from the routine use in pertrochanteric fractures treatment were tested in this study regarding axial forces required to initiate sliding screws dynamization during their transversal load: a third generation Gamma nail (Gamma3® [Stryker, Portage, Michigan]), and two types of extramedullary implants (Selfdynamizable Internal Fixator - SIF [Traffix Ltd, Aleksandrovo, Serbia], one type having three channels for non-cannulated sliding screws of 7 mm radius [SIF-7], and the other type having one channel for the cannulated sliding screw of 10 mm radius [SIF-10]) (Fig. 2). Sliding screw-stem angle (α) was 125° in Gamma3 and 130° in SIF-7 and SIF-10. Pertrochanteric fracture fixation by SIF-7 is mostly performed by using sliding screws, one passing through the upper channel, and the other passing through a one of the two lower channels. Our study included testing of SIF-7 with two sliding screws placed in described manner.

**Fig. 3:**
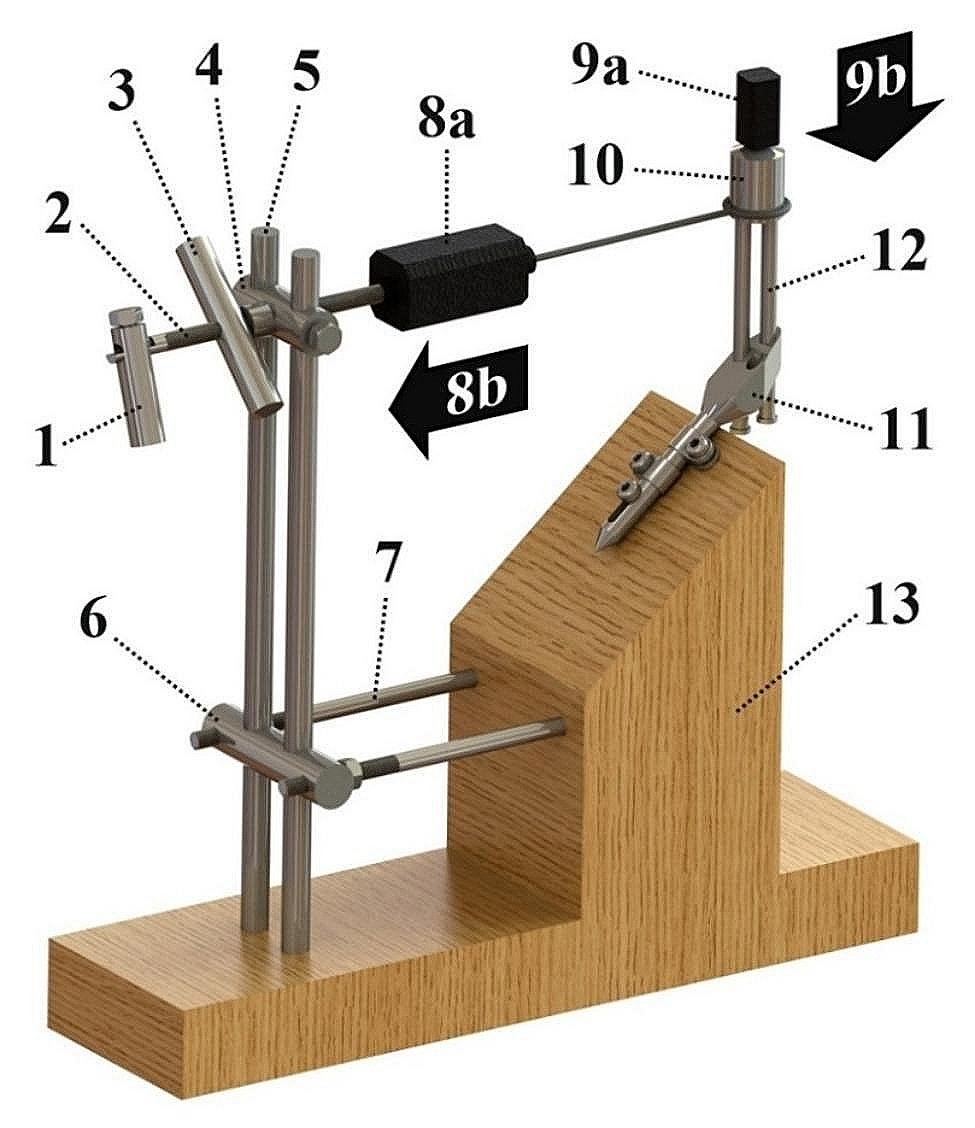
Model of the testing device used in the study. (1) Anti-rotation lever; (2) threaded rod; (3) tension lever; (4) adjustable clamp; (5) vertical bar; (6) supporting clamp; (7) horizontal bar; (8a) portable electronic dynamometer; (8b) direction of the force measured by portable electronic dynamometer (related to Q_t_ in Fig. 1); (9a) integrated electronic dynamometer; (9b) direction of the force measured by integrated electronic dynamometer (related to Q_a_ in Fig. 1); (10) cap; (11) implant stem; (12) sliding screw; and (13) angled block

Each of the implants was attached to the testing device, specially designed for this study, to perform static transversal load of the sliding screws (Fig. 3). This device consisted of the wooden component, in the form of an angled block, and the steel components. The stem of each analysed implant was attached to the angled block, in a position that ensures vertical orientation of the sliding screws with their tips facing upwards. The steel components included two vertical bars fixed to the wooden component, additionally linked to the wooden component by the horizontal bars passed through the supporting clamp. There was an adjustable clamp on the vertical bars too, with the threaded rod passed through. On one side of the treaded rod, the anti-rotation lever was attached, and the tension lever was screwed on the threads. The other side of the threaded rod was attached to a portable electronic dynamometer (Portable electronic scale, Ansenym Guangdong, China). This dynamometer was connected to the sliding screws by a cable. During the testing of SIF-7, containing two sliding screws, the cable was attached to the aluminium cap. This cap had blind holes where sliding screws tips were inserted into and secured by a gypsum mass. While holding the anti-rotation lever, turning of the tension lever was followed by the threaded rod pulling out, thus by transversal load of the sliding screws. The length of a sliding screw between its transversal load level and medial contact point to the implant stem is considered the lever of a sliding screw transversal load, being called the “sliding screw extension”. In SIF-7 sliding screw extension was defined by the lower sliding screw. The feature of the adjusting lever to change its vertical position ensured the sliding screws to be transversally loaded regardless of the extension length. The cap provided two sliding screws to be loaded simultaneously. Electronic dynamometer was used to control static transversal load of the sliding screws (this load is related to Q_t_ in Fig. 1). The testing device was attached to the frame of the tensile testing machine (AGS-X 10kN, Shimadzu, Kyoto, Japan). The moving part of the tensile testing machine contained an integrated load cell. The load cell was in contact with the tip of a sliding screw or the tip of the cap. Downward movement of the load cell, at the rate of 10 millimetres per minute, induced a sliding screws axial load. This axial load is related to Q_a_ in Fig. 1 and it was tracked by the integrated sensor and Trapezium X software, with the possibility of presentation in time dependent graph.

**Fig. 4:**
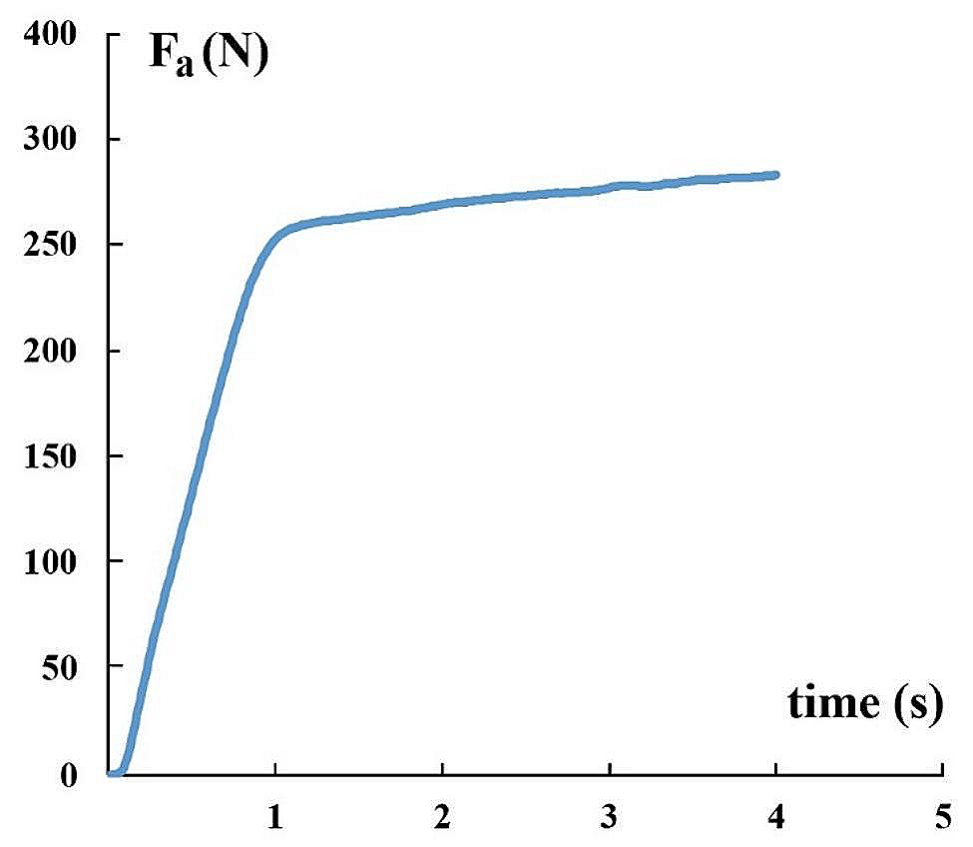
Time-dependent graph of a sliding screw axial load measured during the test. The transition between the first phase (left) and the second phase (right) occurred at 260 N of the sliding screw axial load (F_a_), defining that value as the force required to initiate sliding screw dynamization

Transversal load of a sliding screw passing throw the channel of the implant stem results in a contact between the screw and the channel, being established in two contact points (Fig. 1). Thereby, one contact point is at the lower pole of the medial edge of the channel, and the other contact point is placed on the upper pole of the lateral edge of the channel. Reaction forces, acting at these two contact points transversally to a sliding screw, induce frictional forces that are parallel but in the opposite direction to the axial load. The first phase of the axial loading process described, while the sliding screws are transversally loaded, corresponds to the inital steeply rising part of the time-dependent force graph. The axial load was variable throughout one measurement, due to its constant increase. The first phase of a measurement was static, but the next phase was dynamic, due to the sliding screws dynamization. Compared to the first phase, the graph has a less steep rising slope in the second phase of a measurement (Fig. 4). The point of transition between these two parts of the graph is determined as the moment of sliding screws dynamizing initiation. The product of the lever length (lag screw extension) and the transversal load force is defined as the sliding screw bending moment (BM). The relation between the bending moment (BM) and the axial load required to initiate sliding screws dynamization can be considered as specific for a certain implant.

Sliding screws were loaded by transversal forces of 49 N and 196 N at the level of 20 mm from the tip. Six extensions for Gamma3 sliding screw (every 5 mm in the range of 49–74 mm), and eight extensions for both SIF-7 and SIF-10 sliding screws (every 5 mm in the range of 58–93 mm) were tested in the study. Axial load was repeated and measured four times for each combination of sliding screw extension and transversal load. Axial load required to initiate sliding screws dynamization (AL) was analysed as a function of the bending moment (BM). Using the standard least-squares regression method, this function was presented through the linear regression model AL = a * BM + b, where constant a and constant b determined the slope and the vertical level of the graph. These constants were used to find expected axial loads required to initiate dynamization of the sliding screws for persons of 70 kg body weight. Statistical analysis was performed by IBM SPSS 22 software. Graphs slopes between implants were compared by univariate general linear modelling. Units used were N for AL, and Nm for BM.

## Results

**Fig. 5:**
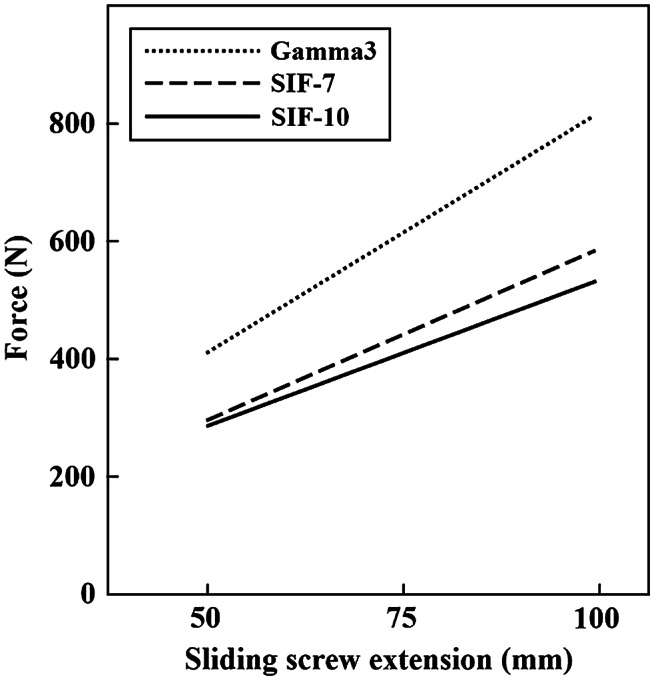
Graph of the forces required to initiate sliding screws dynamization. The forces are presented as a function of 130-degrees angled sliding screws extension in patients with the body weight of 70 kg

Results of the measurements described above approved positive correlation between axial load required to initiate sliding screws dynamization and bending moment of the sliding screws (*p* < 0.05). Higher bending moment was followed by proportionally higher axial load required to dynamize sliding screws. These forces were the highest in Gamma3 and the lowest in SIF-10 implant (Fig. 5; Table 1). The graph slopes were different between any pair of the three implants (*p* < 0.05), being most steep in Gamma 3 and least steep in SIF-10. There was no jamming of the implants during the test performing.

**Table 1:** Regression model parameters

	Regression coefficient		Standard error		r^2^
	a	b	Of coefficient a	Of coefficient b	
Gamma3	32.553	1.312	1.154	10.247	0.945
SIF-10	19.682	39.733	0.653	7.131	0.936
SIF-7	23.257	3.545	1.392	15.199	0.815

Axial load required to initiate sliding screws dynamization (AL) was analysed as a function of sliding screws bending moment (BM) throw the least-squares linear regression method. Regression coefficients a and b defined the relation between BM and expected values of AL. Units used were 1/m for coefficient a and N for coefficient b. Standard errors of a and b are an average range measure of a and b variability. Coefficient of determination (r^2^) represents the degree of variability regarding real AL values in relation to AL values defined throw the regression model.

## Discussion

Considering 70 kg body weight patients (690 N) treated by 130° angled implants (α), results of this study indicate that sliding screws dynamization during the full weight bearing is expected to be realisable in all tested implants (Gamma3, SIF-7, and SIF-10) for shorter femoral necks, but not for all extensions of longer femoral necks. This dynamization is expected to be achievable for higher range of longer femoral necks with SIF-10 than with Gamma3 and SIF-7. Here could be assumed that, in patients with higher body weight and/or larger femoral neck, dynamization of sliding screws can be performed during the full weight bearing sometimes, but more often in other gait phases, or while sitting or lying – when the sliding screws transversal load is lower. Thus, the early full-weight bearing could be preferable in patients with lower body weight and/or shorter femoral neck. Patients with higher body weight and/or longer femoral neck should be suggested to have an early rehabilitation without full-weight bearing, but to introduce it in the later phase of the physical therapy. In an advanced stage of the bone healing process, the load is transferred through the bone more than through the implant, thus the full stance can be allowed in that stage with less risks for the complications related to blocking dynamization. Otherwise, full-weight bearing could perhaps be suggested in early rehabilitation of these patients, but with adduction of the injured leg. Hip adduction results in а decrease of the angle between the axis of resulting hip load (Q_r_) and the sliding screw, thus in decrease of transversal and increase of axial load of the sliding screw.

Higher body weight, longer femoral neck, and smaller sliding screw angle (α) are considered as factors that increase frictional force between the sliding screws and the implant stem, thus potentially as blocking factors in sliding screw dynamization during the full weight bearing gait [7, 18]. Here should be noted that sliding screw extensions used in intramedullary nails are shorter than in extramedullary SIF implants. This is because the stem of the SIF implants is placed completely outside the bone on the lateral femoral side, while the stem of the intramedullary nails is positioned more medially [2, 7, 21]. 

Pauwels’s research into the biomechanics of the hip confirmed that, during the single stance phase of the gait, femoral head bears a load of about three times the body weight, at an angle of 159-degrees to the vertical line [22]. Frankel performed a study with proximal femoral osteotomy and instrumented nail plate fixation, providing in vivo determination of screw force, and found that the load on the implant accounted for about ¼ of the resulting load on the hip [23]. Given these findings, there could be considered that the resulting load on the sliding screws during the single stance phase of gait (Q_r_) is ¾ of the body weight.

Loch et al. analysed the forces required to initiate sliding of lag screws for three types of second-generation intramedullary nails (Gamma, Recon and ZMS), IMHS and SHS, performing transversal load using static weights [7]. Their results for second-generation Gamma nail were similar as results for third-generation Gamma nail in our study. Both the studies indicate that, during the single stance phase of the gait, dynamization of the Gamma nails sliding screws is expected to be achieved for lower bending moments, but not for higher bending moments.

Gamma3 and SIF-10 implants are comparable due to the similar radius of the sliding screws (Gamma3 sliding screw is just 0.5 mm wider than in SIF-10) [24]. In our study, initial dynamizing forces were found to be lower in SIF-10 than in Gamma3. This could be influenced by the contact surface between the sliding screw and the channel of the implant stem. Channel surface is continual in SIF implants, while it is interrupted in Gamma Nails (due to the central axial cavity of the stem) [7], making it smaller than in SIF-10. Furthermore, there is a larger contact surface if the stem cross section is quadrangular, like in SIF implants, than if it is circular, like in Gamma nail. Loch et al. also found that а larger contact surface between the stem and the sliding screw is followed by lower forces required to initiate the sliding screw dynamization [7]. 

Coefficient of determination was lower in SIF-7 than in Gamma 3 and SIF-10. Every sliding screw has its own range of variation for forces required to initiate its dynamization. Thus, comparing to one sliding screw implants, two sliding screws implants could be considered to have higher variation described.

In addition to the points of contact between sliding screws and stem, there are other factors that influence the dynamization of pertrochanteric fractures. Contact between the stem of an intramedullary nail and the proximal pertrochanteric fracture fragment can be considered a potentially blocking factor in the dynamization. This thesis is supported by the results of Matre et al. who found higher rate of reoperations with intramedullary nailing than with extramedullary SHS fixation of two-part pertrochanteric fractures [25]. Jones et al. also found a higher reoperation rate in the treatment of stable trochanteric fractures with intramedullary nails, than with SHS [26]. On the other hand, excessive dynamization of trochanteric fractures, followed by excessive shortening of the femoral offset, should be prevented [27, 28]. Excessive dynamization of an extramedullary fixed pertrochanteric fracture could theoretically be found more with SHS or SIF-7, due to the higher range of sliding screws dynamization, than with SIF-10 which contains a mechanism to prevent an excessive dynamization (Fig. 2).

The experiment of this study included a fixed-point loading design. If distributed loading is to be achieved, a skeletal specimen or 3D model study should be required.

In conclusion, larger contact surface between the sliding screws and the implant stem is associated with lower forces required to initiate sliding screws dynamization. These forces are higher if the bending moment of the sliding screws is higher. After internal fixation of a pertrochanteric fracture, patients with longer femoral neck or higher body weight could have different programme of early postoperative rehabilitation than lighter patients or patients with shorter femoral neck.

## Data Availability

The datasets used and analysed during the current study available from the corresponding author on reasonable request.

## Abbreviations

AL: Axial load required to initiate sliding screws dynamization
BM: sliding screws bending moment
SIF: Selfdynamizable Internal Fixator
